# Sub-parts-per-trillion level sensitivity in trace gas detection by cantilever-enhanced photo-acoustic spectroscopy

**DOI:** 10.1038/s41598-018-20087-9

**Published:** 2018-01-30

**Authors:** Teemu Tomberg, Markku Vainio, Tuomas Hieta, Lauri Halonen

**Affiliations:** 1 Department of Chemistry, University of Helsinki, P.O. Box 55, FI-00014 Helsinki, Finland; 2 Laboratory of Photonics, Tampere University of Technology, Tampere, FI-33101 Finland; 3 Gasera Ltd., Lemminkäisenkatu 59, FI-20520 Turku, Finland

## Abstract

An exceptional property of photo-acoustic spectroscopy is the zero-background in wavelength modulation configuration while the signal varies linearly as a function of absorbed laser power. Here, we make use of this property by combining a highly sensitive cantilever-enhanced photo-acoustic detector, a particularly stable high-power narrow-linewidth mid-infrared continuous-wave optical parametric oscillator, and a strong absorption cross-section of hydrogen fluoride to demonstrate the ability of cantilever-enhanced photo-acoustic spectroscopy to reach sub-parts-per-trillion level sensitivity in trace gas detection. The high stability of the experimental setup allows long averaging times. A noise equivalent concentration of 650 parts-per-quadrillion is reached in 32 minutes.

## Introduction

In molecular trace gas analysis, researchers are constantly pushing towards more sensitive, affordable and robust instruments as required by the industry and the scientific community. Modern photo-acoustic spectroscopic (PAS) techniques, such as cantilever-enhanced photo-acoustic spectroscopy (CEPAS)^1,2^, quartz-enhanced photo-acoustic spectroscopy (QEPAS)^3,4^ and variants of cavity-enhanced photo-acoustic spectroscopy^5,6^, are in the leading edge of this progress as they meet the criteria^7^. As an example, an impressive detection limit of 750 parts-per-quadrillion (ppq) of SF_6_ in ultrapure Ar was recently demonstrated by Xiong *et al*.^8^ using a resonant piezo-electric crystal detector similar to QEPAS. Peltola *et al*.^9^ reported another noteworthy result, where a noise equivalent detection limit of 50 parts-per-trillion (ppt) of NO_2_ in ambient air was demonstrated using CEPAS.

There are several advantages in photo-acoustic techniques. They combine wavelength independent operation with a low sample gas volume, a zero-background signal and a high detection sensitivity that is, as a first approximation, proportional to the incident laser power^7^. A PA signal is produced by non-radiative relaxation of periodically excited molecules, conventionally detected by a type of microphone. The signal magnitude, denoted by *S*, is conveniently expressed in millivolts as:

1 $$S={S}_{m}P\,{C}_{cell}{N}_{tot}{c}_{m}\sigma $$

where *S*_*m*_ is the microphone sensitivity in mV/Pa, *P* is the optical power of the incident light in W, *C*_*cell*_ is the PA cell response constant in Pa/cm^−1^W, *N*_*tot*_ is the total number density of molecules in molecule/cm^3^, *c*_*m*_ is the concentration given as volume mixing ratio, and *σ* is the absorption cross section in cm^−1^ cm^3^/molecule. It is self-evident that in order to measure the smallest concentration *c*_*m*_, one needs to maximize the other terms in the equation. For CEPAS, the *S*_*m*_ term is one of the highest available. In CEPAS, the pressure variations inside the PA cell are detected by measuring the movement of a silicon cantilever by a laser interferometer to a picometer resolution^1,2^. The cantilever operates best in a non-resonant mode, in a frequency range from 10 to 100 Hz as the signal to noise ratio (SNR) does not increase by operating at the cantilever resonance frequency^2^. Next, the cell response *C*_*cell*_ may be increased by using an acoustical resonance of the PA cell. The term *P* is maximized by choosing a high power laser light source and possibly using a multi pass or a cavity-enhanced configuration^5,6,10^. A strong absorption feature of the molecule of interest should be chosen to maximize *σ*, while simultaneously paying attention to availability of suitable lasers for the wavelength in question, and to interference from other absorbing species. Lastly, the pressure, or *N*_*tot*_, should be optimized for spectroscopy, for example, in terms of the absorption feature linewidth.

In this article, we demonstrate the ability of CEPAS to reach a sub-ppt level sensitivity in trace gas detection, by making use of the linear scalability of the photo-acoustic signal by absorbed optical power. More specifically, we combine a sensitive CEPAS detector, a particularly stable high-power narrow-linewidth optical parametric oscillator (OPO), and a strong absorption cross-section of hydrogen fluoride (HF), to reach a noise-equivalent concentration (NEC) of 650 parts-per-quadrillion (ppq) by averaging for 32 minutes. The result, to our knowledge, is the lowest concentration ever reported for CEPAS, the highest HF sensitivity ever reported for a laser-based technique^11,12^, and one of the few laser-based gas analyzers capable of sub-ppt level sensitivity in trace gas detection^8,13,14^. Detection of HF at ultra low concentrations is needed in, for example, registering of hazards at work sites^15^. Hydrogen fluoride, a highly reactive and corrosive gas used widely in industry, is hazardous to health already at the concentration of 3 parts-per-million (ppm)^16^. To vegetation, even a concentration of few parts-per-billion (ppb) has been shown to be harmful^17^. So far, the highest HF sensitivity using laser absorption spectroscopy has been achieved in the work by Craig *et al*.^11^ where they report a NEC of 38 ppt in 1 s, whereas in our experiment a NEC of 5 ppt is achieved in 1 s.

## Results

### Experimental setup

**Figure 1:**
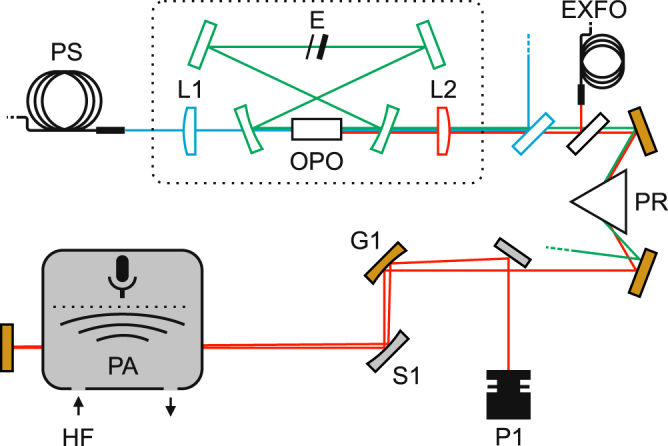
Schematic illustration of the experimental configuration. PS: pump laser source, L1: lens with *f* = 200 mm, L2: lens with *f* = 150 mm, E: two etalons, EXFO: wavemeter, PR: equilateral dispersive prism, G1: gold coated mirror with *R* = 200 mm, S1: silver coated mirror with *R* = 50 mm, P1: power meter, PA: photo-acoustic analyzer, HF: hydrogen fluoride gas in dry air.

The novelty of the experimental setup, shown schematically in Fig. 1, lies in a tailored OPO light source and a clever usage of high-sensitivity-allowing sub-systems. The strongest absorption lines of HF are located around 2476 nm, which we are able to access with the special high-power narrow-linewidth optical parametric oscillator that produces coherent light at three principle wavelengths: a 1064 nm pump beam, a 1866 nm signal beam and a 2476 nm idler beam. Therefore, the OPO operates in a difficult wavelength region close to degeneracy and in the presence of strong water absorption for the resonant signal wavelength, severely hampering a stable single mode operation^18,19^. Despite that, the long term stability of the OPO was improved by almost a factor of 100 over the previously reported OPOs^20^, which was crucial in terms of reaching the exceptional trace gas sensitivity via long averaging times. The stability problems were solved by first placing the cavity in an enclosure continuously purged with dry air. The purging reduced the humidity inside the enclosure to about one tenth of normal ambient air. This diminished the observed power fluctuations and increased the output power as the cavity losses were reduced. Second, two uncoated YAG etalons (0.3 and 2 mm thick) were placed inside the cavity at the secondary focal point to narrow the OPO gain bandwidth. The two etalons, with designed non-matching FSRs, were rotated to allow maximum transmission at a point of low water absorption near 1866 nm. In this way, the cavity losses and gain bandwidth were further reduced because of the surrounding stronger water absorption. With the help of these two methods, the signal wavelength was stabilized, and a single mode operation of several hours with up to 950 mW output power at 2.5 μm with 50% conversion efficiency was achieved. For lower power levels in the range of 100 to 600 mW, a single mode operation for even longer periods was possible.

As for more technical details, the singly resonant continuous-wave (CW) OPO was otherwise built using the same design principles as described in previous articles^20–22^: The OPO is pumped by a continuous-wave distributed feedback (DFB) laser (Eagleyard DFB:1064-0040-BFY02-0002) operating at 1064 nm and amplified by an ytterbium fiber amplifier (IPG YAR-20k-1064-LP-SF) to the maximum optical output power of 20 W. The pump beam, after a beam expansion, is focused into the OPO cavity with an anti-reflection coated lens. In the OPO, the pump beam is focused into a 50 mm-long MgO-doped, periodically poled lithium niobate (MgO:PPLN by HC Photonics) crystal, the poling period of which ranges from 26.5 to 32.5 μm in a fanout pattern. The crystal is placed on an aluminum holder, the temperature of which can be stabilized anywhere between 20 and 100 °C with a precision of 100 mK using thermo-electric coolers and a commercial controller (Newport 350B). A bow-tie ring cavity is built around the crystal. The two plano-concave dielectric mirrors have a 125 mm radius of curvature, high transmission coatings for the pump and idler wavelength and a high reflectance for the signal wavelength. The two plane mirrors of the cavity possess the same coatings.

The idler beam is separated from the residual pump and signal beams using a combination of a dichroic mirror and an equilateral dispersive prism (Thorlabs PS853). About 4% of the idler and signal beams is sampled by a wedged uncoated CaF_2_ window to an EXFO WA-1500 wavemeter for monitoring purposes. After the beam separation, the idler beam is guided through a cell of a PA detector in a double-pass configuration. The optical power after the cell is measured with a power meter. The cantilever-enhanced photo-acoustic detection system, manufactured by Gasera Ltd., is equipped with a PA cell of 95 mm in length, 4 mm in diameter and 7 ml in total volume. The cell has a gold coated surface and CaF _2_ windows with anti-reflection coating for the idler wavelength (Thorlabs WG50530-D). The sample gas pressure in the cell is regulated to 200 mbar for optimal spectral linewidths, and the temperature to 50 °C by an automated control system. The periodic 30 Hz photo-acoustic signal is formed by sinusoidal wavelength modulation of the OPO pump laser with a 1.1 GHz modulation amplitude. A lock-in detection scheme is employed to perform a second harmonic signal detection. Use of wavelength modulation effectively eliminates the background PA signal arising from the windows and walls of the PA cell. The modulation frequency was optimized by measuring the acoustic noise spectrum of the CEPAS sensor and identifying an interference free region (see Supplementary Figure 1).

### Signal verification

Photo-acoustic wavelength modulation spectroscopy was performed on a strong ro-vibrational transition of HF centered at 2475.8836 nm with a line intensity of 2.381 × 10^−18^ cm^−1^/(molecules cm^−2^) at 296 K^23^. The wavelength in question is good for selective spectroscopy since it is almost free of spectral interference from other atmospheric gases as expected by simulations and confirmed by measurements. In a normal operation mode, only the peak of the second harmonic signal of the HF absorption line needs to be measured in order to determine the HF concentration. Although the method is fast, it is blind to possible fringes and interfering absorption visible in full spectral scans. These error sources have to be pre-examined before the experiment and predicted during the experiment with the help of additional measurements as necessary.

**Figure 2:**
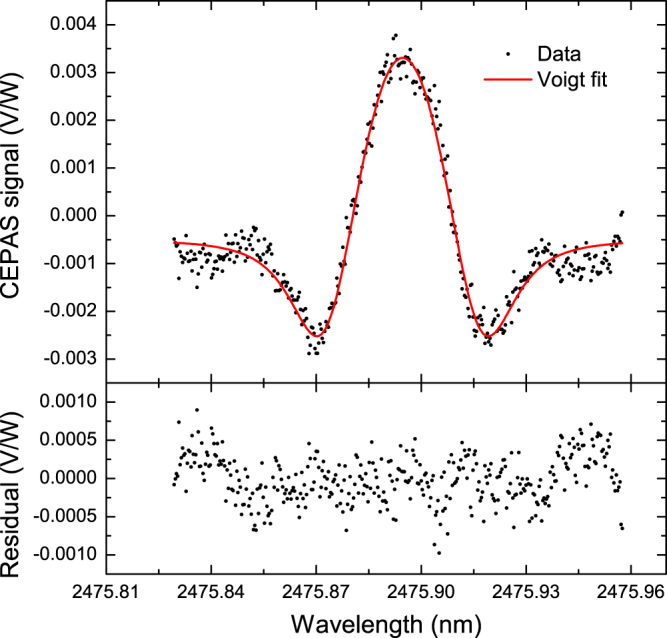
The upper figure shows a second harmonic signal of 97 ppt HF in dry air, measured with an optical power of 740 mW, and a least squares fit of the second harmonic signal of a Voigt profile to the data. The lower figure shows the residual after subtraction of the fit. The background offset is attributed to tails of nearby water absorption, the water concentration being 1010 ppm.

In order to identify the HF line and possible background signals, the full spectrum is first recorded once, and then repeated if experimental parameters, such as pressure, have been changed. Fig. 2 shows a recorded second harmonic spectrum of 97 ppt of HF. Black dots represent the measurement data recorded in about 14 minutes for the densely sampled spectrum. A least squares second harmonic Voigt fit^24^ (red curve) and its residual (lower figure) show a good match to the data. The obtained line parameters, including the Lorentzian linewidth and modulation amplitude (MA), agree well with expectations. The modulation amplitude was included as a fit parameter to verify its value, which was adjusted to minimize residual amplitude modulation (RAM) in the second harmonic signal. The RAM was caused by an etalon effect in the PPLN crystal having non-optimal anti-reflection coatings for the idler beam. The RAM couples to the CEPAS signal through absorption in the windows of the PA cell, a common problem in photo-acoustics^7^. Simulations, based on the measured FSR of the PPLN crystal, were used to find an optimal modulation amplitude, for which the second harmonic signal of the RAM would disappear^25,26^ (see the Supplementary Note 1 for more details). As a result, the RAM was reduced to an amplitude level equal to HF at the concentration of 8 ppt, as seen on the tails of the residual in Fig. 2. The improvement was more than ten-fold, eliminating RAM from the sensitivity limiting factors of the system as the residual RAM was found to be stable and therefore does not contribute to the noise signal even at long averaging times. A simple further reduction to the RAM signal would be possible by using more optimized anti-reflection coatings of the PPLN crystal, or Brewster cut ends.

As for interfering absorption features, the most significant interferer is a water line centered at 2475.72 nm. Simulations using the HITRAN 2012 database^23^ demonstrate that the contribution of typical ambient water concentration of 1.4% at 200 mbar pressure creates a 30 times stronger second harmonic background signal than HF at a concentration of 1 ppt. A solution is to reduce the pressure, stabilize and closely monitor the water concentration, or reduce it to an insignificant level compared to the sensitivity of the experiment. For the experiments reported here, we continuously measured the water concentration using a Vaisala DMT143 dewpoint transmitter. For the spectrum shown in Fig. 2, the amount of water in the sample was 1010 ppm and the background offset is 15% relative to the peak, agreeing qualitatively with the simulations. There is a further reason to monitor the humidity because we noticed that the HF concentration in the sample cell depends almost linearly on the H_2_O concentration in the carrying gas on a short time scale (see Supplementary Figure 3). The dependence was not because of changes in the background level due to water, but most likely because amount of HF transferred to the measurement cell was varied due to adsorption/desorption chemistry between H_2_O and HF.

### Calibration

**Figure 3:**
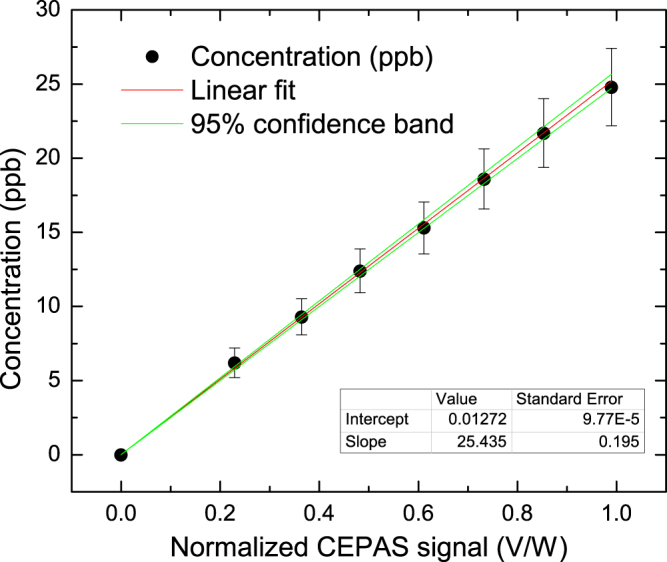
CEPAS signal as a function of HF concentration and a linear fit to the data. The measurement was performed using an optical power of 740 mW. Uncertainties of the MFCs and the concentration of the sample bottle are included.

Photo-acoustic spectroscopy measures directly the absorption of light, rather than, for example, calculating it from the attenuation of light relative to a background, as is the case in more common transmission spectroscopy. Therefore, photo-acoustic analyzers always require calibration by referencing to a known sample concentration. The data points for the calibration were collected in two steps. First, the power-normalized CEPAS signal was measured at different controlled HF concentrations. Second, the zero HF point was determined as the background signal level of a spectral scan in Fig. 2. A linear least squares fit to the data, weighted by the uncertainty of the data points, was then determined as the response curve of the CEPAS system. The result, shown in Fig. 3, confirms a linear response of the system (*R*^2^ > 0.999). The observed offset, accounted by the calibration, is caused by interfering absorption of a nearby water transition, as predicted by the simulations. Uncertainty in the produced analyte concentration, illustrated by the error bars, take into account the accuracy of the mass flow controllers (MFC) used in sample preparation for ppb level concentrations, and the uncertainty of the sample bottle concentration. Another source of measurement error was adsorption of HF, which has to be carefully acknowledged for reproducible measurements. In the case of highly adsorptive molecules, such as HF, it is easier to remove than to enrich molecules on a surface^27^. Therefore, before the calibration measurement, the adsorption was first saturated to a high level by flowing the sample gas for more than ten hours on a concentration level slightly higher than the first measurement level. The HF concentration was then dropped stepwise and adsorption was allowed to stabilize before taking a CEPAS signal reading.

### Sensitivity

**Figure 4:**
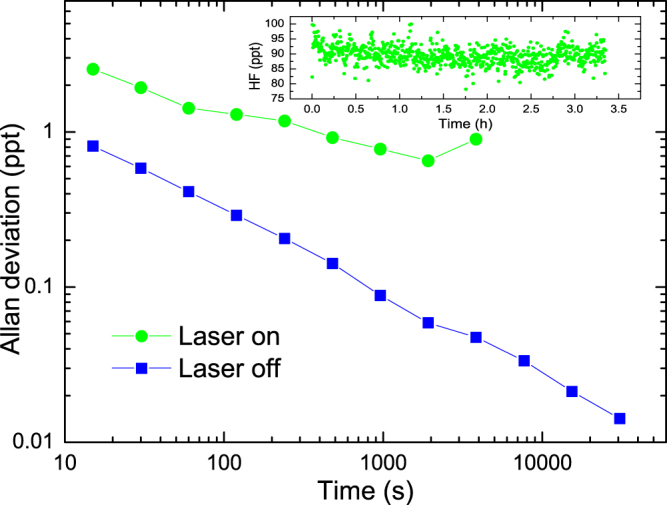
Allan deviation of the HF volume mixing ratio as a function of averaging time. The inset shows the measurement data, obtained with an optical power of 950 mW. The slopes of the ‘laser on’ and ‘laser off’ Allan deviation traces are −0.28 and −0.5, respectively.

The stability and sensitivity of the PA system was investigated using the Allan deviation^28^. The CEPAS signal was measured with a high optical power of 950 mW at the HF concentration of 92 ppt. For a reference, the PA signal was also measured when the laser was turned off to show that mechanical or acoustical noise is not affecting the performance. The main results are presented in Fig. 4 and additional details in Supplementary Figure 3. The inset in Fig. 4 shows the data used for the Allan deviation plot with the laser on. The Allan deviation is used to determine NEC of the system^28^. A noise equivalent concentration of 2.5 ppt was reached in one gas exchange cycle (15 s), and 0.65 ppt in 32 minutes. A normalized noise equivalent absorption (NNEA) coefficient of 5.19 × 10^−10^ W cm^−1^ Hz^−1/2^ was reached with a sampling time of 15 s and an optical power of 950 mW. The achieved sensitivity is decreased by the about 40% gas exchange/measurement duty cycle. If the measurement was operated with a 100% duty cycle, i.e., without any gas exchange, it is possible to obtain PA signal readings at a 1 Hz rate and a lower NEC of 5 ppt in 1 s corresponding to NNEA of 2.7 × 10^−10^ W cm^−1^ Hz^−1/2^ was achieved. However, without the gas exchange, the system is unstable in a long term because in few minutes the desorption of HF from the walls of the PA cell will cause the HF concentration to drift until a new unknown steady state is reached.The NNEA matches those reported using similar CEPAS^2,9^, compares favorably with most high-end photo-acoustic analyzers^6,8,29,30^, but loses an order of magnitude to the best ones reported^5,31^.

## Discussion

**Figure 5:**
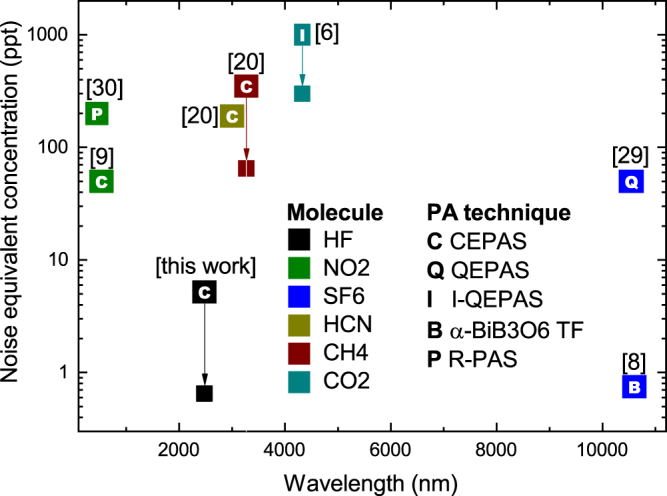
Ranking graph of noise equivalent concentrations for photo-acoustic techniques, expressed in ppt and normalized to 1 s detection time and averaging indicated by an arrow if reported. The NECs (up to 1 ppb) are related to 5 different techniques (letter) and 6 different molecules (color). References are given in brackets. See Supplementary Table 1 for additional information of these results.

The results presented in this article demonstrate a great potential of photo-acoustic spectroscopy, and especially CEPAS, for remarkably sensitive and selective trace gas analyzers. Figure 5 highlights this by comparing the achieved noise equivalent concentrations of the best photo-acoustic experiments in the literature. The detection time is normalized to 1 s for comparison and averaging indicated by an arrow if reported (more detailed information provided in Supplementary Table 1). Half of the reported NECs below 1 ppb level have been achieved with CEPAS and the other half with QEPAS or its variants. The comparison is taken in concentration instead of, for example, NNEA to emphasize the importance of the entire experiment, including the accessible wavelength range, stability of the light source, absorption path length and photo-acoustic relaxation effects.

In our present setup, the stability was primely limited by fluctuations in the produced HF concentration. The results (see Supplementary Figure 3) show clear correlation between small changes, in a time scale of few minutes, of the measured H_2_O and HF concentration, unattributable to changes in the background signal level by tails of nearby water absorption line. The correlation is believed to be caused by water affecting the amount of HF carried to the measurement cell by competing adsorption on the surfaces. Although such water induced changes in the HF level are rapid, the transition to a steady HF level is slow especially with small HF concentrations. In general to reduce such effects and increase the response time of the system, one can resort to an open cell design and high flow rates as in Ref.^11^. To implement such a design using photo-acoustic detection, one could make use of a differential photo-acoustic detection scheme^32^. A suitable choice of gas pipe material may also have a positive effect on the response time^27^. However, sampling was not in the main focus of our research but demonstrating realistic results of the achievable HF detection sensitivity. Unlike in many of the other works cited in Fig. 5, we conducted the experiment in close to a realistic gas matrix, allowing interference, such as the HF/H_2_O interplay, to be observed. A future research could include, for example, development of a more rapid sampling system. To estimate the performance with an optimized sampling system, we assume in that case the noise type to be white and the slope of the Allan deviation therefore to be -0.5. Taking 5 ppt at 1 s averaging time as our benchmark, at 15 s averaging the noise equivalent concentration would be 1.3 ppt and at 32 min only 119 ppq. The remaining difference between the predicted and measured ‘laser off’-noise of 530 ppq at 15 s, for example, could then be attributed to the noise of the light source (see Supplementary Figure 3). Optimizing the sampling system could also include increasing the gas exchange/PA measurement duty cycle, which would also lead to better overall sensitivity. Finally, it is worth noting that with the emergence of new high power semiconductor diode lasers, there is potential to even improve the results presented in this article and simultaneously make the equipment capable to field measurements.

## Methods

### Sample gas preparation

Two mass flow controllers (*Aera* and *MKS Instruments*) were used to prepare a sample gas of desired HF concentration by mixing 3.05 ppm of HF (AGA) and dry ambient air (humidity around 1050 ppm). Flow rate ranges were 4–200 and 40–2000 sccm for the MFC controlling HF and air, respectively. The controlled HF mixing ratios were then in the range of 6 to 400 ppb. Lower stable concentrations on the order of 100 ppt were enabled by the slow desorption of HF in the sampling system while no HF was fed to the gas flow.

### Automated gas exchange system

Two mass-flow-controllers create a single continuous by-pass flow out of which the automated gas exchange system pumps the gas into the PA cell. Every 15 s the cell valves are switched to direct the flow through the PA cell. After a few seconds, the input valve is closed while the exhaust pump continues to reduce the cell pressure. In about 3 s the target pressure is reached, all cell valves are closed and the photo-acoustic measurement is executed. A total 6 s for each 15 s cycle is available to record the PA signal via a USB data link at a 1 Hz sample rate. The set of six samples is then averaged to form one measurement point.

### Calculation of NNEA

The normalized noise equivalent absorption (W cm^−1^ (Hz)^−1/2^) coefficient can be determined using the following equation:

2 $$NNEA={\alpha }_{min}\,P\,\sqrt{t},$$

where *α*_*min*_ = *α*/*SNR* (cm^−1^) is the noise equivalent absorption coefficient, which can be calculated using the HITRAN database (http://www.hitran.com) when the targeted wavelength, SNR, trace gas pressure, and temperature are known. In our work, we approximated the line shape by a pseudo Voigt function^33^. The quantity *P* (W) is the incident optical power and *t* (s) the measurement time. Because our sensitivity did not improve as square root of the measurement time, we chose the shortest measurement time of 15 s for the calculation. Hence, the NNEA = 5.2 × 10^−10^ W cm^−1^ (Hz)^−1/2^ can be calculated if *α*_*min*_ = 1.4 × 10^−10^ cm^−1^ and *P* = 0.95 W. For a measurement without gas exchange, the short term stability was even better and an NNEA of 2.7 × 10^−10^ W cm^−1^Hz^−1/2^ was reached with *α*_*min*_ = 2.9 × 10^−10^ cm^−1^ and *t* = 1 s.

### Data availability

The authors declare that all data supporting the findings of this study can be found within the article and its Supplementary Information Files. Additional data supporting the findings of this study are available from the corresponding author (L.H.) upon reasonable request.

## Electronic supplementary material

Supplementary information
