# Supplementary material for: Sub-parts-per-trillion level sensitivity in trace gas detection by cantilever-enhanced photo-acoustic spectroscopy
Source: Sci Rep. 2018 Jan 30;8:1848. doi: 10.1038/s41598-018-20087-9 (PMC5789827; doi:10.1038/s41598-018-20087-9)
Supplement: Supplementary file 1 — Supplementary information [file 41598_2018_20087_MOESM1_ESM.pdf]

Supplementary Information

Sub-parts-per-trillion level sensitivity in trace gas detection by cantilever-enhanced photo-acoustic spectroscopy

Teemu Tomberg[1], Markku Vainio[1,2], Tuomas Hieta[3], and Lauri Halonen[1,*]

[1]Department of Chemistry, University of Helsinki, P.O. Box 55, FI-00014 Helsinki, Finland
[2]Laboratory of Photonics, Tampere University of Technology, FI-33101, Finland
[3]Gasera Ltd., Lemminkäisenkatu 59, FI-20520 Turku, Finland
[*]Corresponding author: lauri.halonen@helsinki.fi

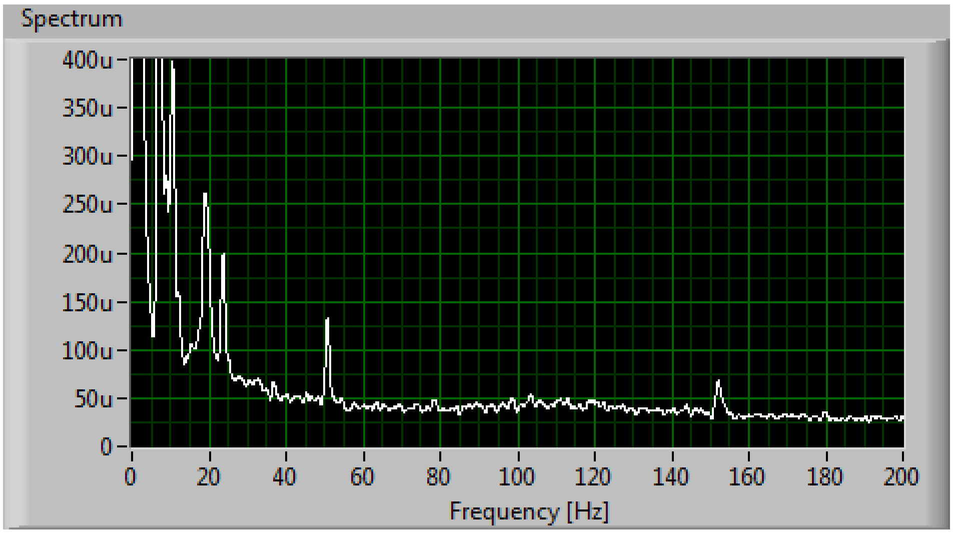

Supplementary Figure 1: Acoustic noise spectrum of the cantilever-enhanced photo-acoustic detector. The spectrum was recorded by sampling the cantilever position at 5.2 kHz sampling rate, calculating a fast Fourier transform of 1.57 s block and averaging 50 spectra. As seen in the figure, the chosen 30 Hz modulation frequency, and its second harmonic, are free from spurious noise signals. The vertical axis is expressed in µV.

Supplementary Note 1: Simulation of the optimal modulation amplitude for WMS.

According to the manufacturer, the reflectance of the end faces of the PPLN crystal is about 15% for the idler wavelength. This is enough to result in clear nonlinear amplitude modulation of the idler beam, as it is wavelength modulated for photo-acoustic spectroscopy. The amplitude modulation is imprinted on the photo-acoustic signal severely distorting it without further measures. The problem was solved by optimizing the wavelength modulation amplitude (WMA) with respect to the residual amplitude modulation as explained below.

The optimization began by performing spectroscopic simulations, using the HITRAN 2012 database, to determine the optimum WMA at a specific pressure. We were limited by tuning range of the pump laser to maximum WMA of 0.04 cm^{-1}. The highest pressure, for which this WMA corresponds the peak of the second harmonic HF signal, was found to be 200 mbar with tolerance of ±0.01 cm^{-1} for 5% change in the peak value. The 0.04 cm^{-1} WMA is close to the FSR of the etalon, measured to be about 0.046 cm^{-1}. Because the PA signal caused by the RAM was noted to depend on the WMA, a simulation was performed to find a WMA for which a specific Fourier-component of the RAM would go to zero independent of idler center wavelength. The etalon transmission was modeled using the Airy function

$$T = \frac{1}{1 + F\sin^2\left(\frac{K}{2}\right)} \tag{1}$$

$$F = 4\frac{R^2}{(1-R^2)^2} \tag{2}$$

which, for a small reflectance of 15%, is close to the sinusoidal modulation function

$$\nu = \nu_0 + \nu_{off} + A\sin(2\pi t f_{mod}) \tag{3}$$

In the simulation, the modulation function (3), with a frequency f_{mod} = 30 Hz, and as a function of time t, was fed to the etalon transmission function (1) as

$$K = \frac{2\pi\nu}{FSR} \quad (4)$$

The optical frequency offset ν_{off} was varied over range of one FSR to cover all possible positions of modulation with respect to the etalon. Fast Fourier transform was calculated for each of the steps and the magnitudes of the *F*-components of interest were noted. Finally, the procedure was repeated for different wavelength modulation amplitudes A to identify minimums of certain *F*-components. The result is depicted in Supplementary Figure 2. Noting the maximum allowed WMA, we find out that the WMA, for which the second harmonic (60 Hz) of the RAM goes to zero is 0.817 times the FSR being to about 0.037 cm^{-1}. This was determined as the optimal WMA for this application with little reduction in the second harmonic PA-signal strength. In practice, the limitation of reduction of RAM by this method was found to depend on how carefully one can find the optimal WMA and how closely the modulation waveform matches the etalon transmission function.

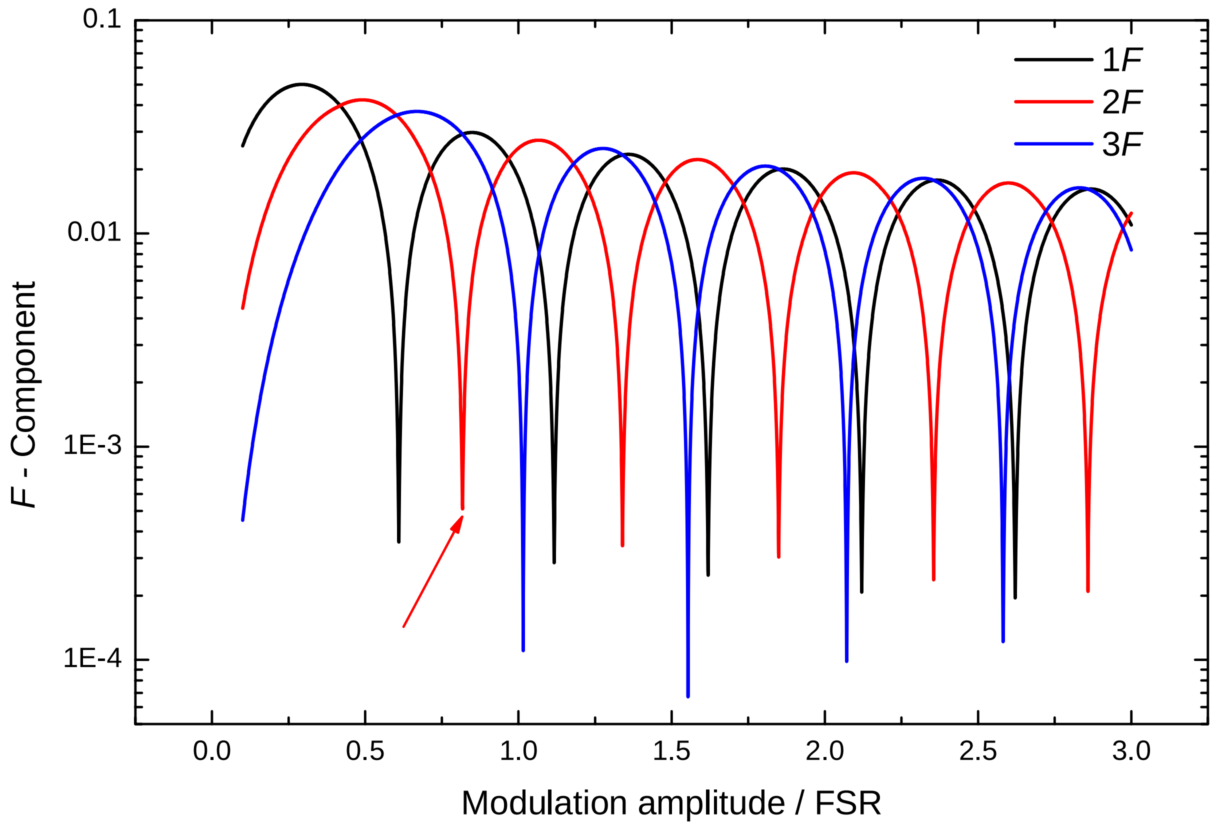

Supplementary Figure 2: Simulation of magnitude of RAM Fourier-components as a function of wavelength modulation amplitude. The unit of the vertical axis is arbitrary. The horizontal axis represents the WMA relative to the FSR of the etalon causing the RAM. The first minimum for 1f-component is found at 0.611×FSR and the first minimum for 2f-component at 0.817×FSR

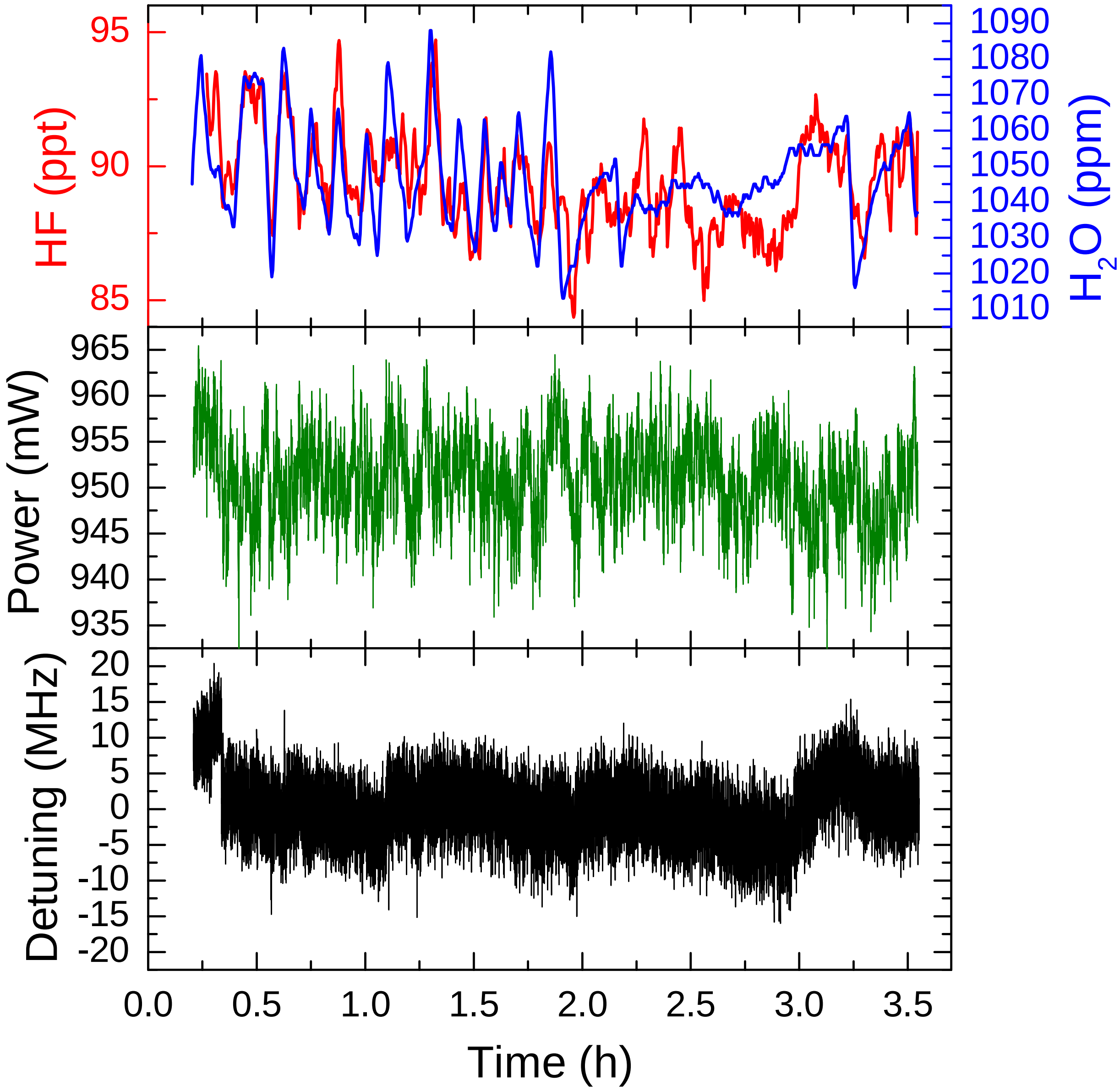

Supplementary Figure 3: Measured water and HF concentration of the sample gas in addition to the wavelength and optical power of the OPO idler beam. The red trace shows the same measurement of HF concentration as in the inset of Figure 4 of the main article, but with 1.5 min moving average filter applied. The blue trace shows the measured water concentration of the same sample gas. The water concentration was measured using a Vaisala DMT143 dewpoint transmitter connected to the dry air feed before mixing with HF in N_2. The green trace shows the optical power of the OPO light source and the black trace the wavelength detuning from the line center measured with an EXFO WA-1500 wavemeter.

Supplementary Table 1: Ranking table of noise equivalent concentrations. Additional information to Figure 5 of the main article.

Ref	Mol.	NEC in 1s	NEC by Avg	Method	Wavelength	Power	NNEA	Matrix
	HF	10 ppt	650 ppq@32min	CEPAS	2475 nm	0.95W	5.2E-10	Dry air
[9]	NO_2	50 ppt	-	CEPAS	532 nm	4.7W	2.6E-10	Dry air
[8]	SF_6	750 ppq	-	α-BiB_3O_6 tuning fork	10600 nm	1W	1.62E-10	Ar
[29]	SF_6	50 ppt	-	QEPAS	10540 nm	18mW	2.7E-10	N2
[20]	HCN	190 ppt	-	CEPAS	3002 nm	0.6W	1.8E-9	N_2
[20]	CH_4	350 ppt	65 ppt@30s	CEPAS	3271 nm	0.6W	1.8E-9	N_2
[6]	CO_2	1000 ppt	300 ppt@20s	I-QEPAS	4327 nm	0.72	3.2E-10	Air
[30]	NO_2	200 ppt	-	R-PAS	444 nm	333mW	6.66E-10	N_2
